# Supplementary material for: The complete genome sequence of the crayfish pathogen Candidatus Paracoxiella cheracis n.g. n.sp. provides insight into pathogenesis and the phylogeny of the Coxiellaceae family
Source: mSphere. 2025 Mar 10;10(4):e01002-24. doi: 10.1128/msphere.01002-24 (PMC12039232; doi:10.1128/msphere.01002-24)
Supplement: Legends for supplemental material — Legends for Files S1, S2, and S3. [file msphere.01002-24-s0004.docx]

**Supplemental material Legends**

**Supplementary File 1. Workflow for quality controls, kraken assignment and genome assembly.** Markdown document with individual commands used on the long read data for quality controls, kraken assignment and genome assembly.

**Supplementary File 2. The Kraken2 classification of the chromosomal reads using a GTDB-based database.** Kraken2 report describing taxonomic classification of chromosome-only long read data.

**Supplmentary File 3. Predicted Dot/Icm effector proteins based on Bastion4 and T4Sepp tools.** IS elements are highlighted in blue/purple, predicted effectors with no significant similarity found are highlighted green and proteins predicted to be effectors using both tools are shown in orange. For each predicted effector, the most similar protein identified using BLASTp is listed with both the percentage of sequence coverage and percentage identity.
